# Associations between active commuting and physical activity in working adults: Cross-sectional results from the Commuting and Health in Cambridge study

**DOI:** 10.1016/j.ypmed.2012.08.019

**Published:** 2012-11

**Authors:** Lin Yang, Jenna Panter, Simon J. Griffin, David Ogilvie

**Affiliations:** MRC Epidemiology Unit, Institute of Metabolic Science, Box 285, Addenbrooke's Hospital, Cambridge, CB2 0QQ, UK; UKCRC Centre for Diet and Activity Research (CEDAR), Institute of Public Health, Forvie Site, Robinson Way, Cambridge, CB2 0SR, UK

**Keywords:** Accelerometry, Active commuting, Physical activity, Working adults

## Abstract

**Objective:**

To quantify the association between time spent in active commuting and in moderate to vigorous physical activity (MVPA) in a sample of working adults living in both urban and rural locations.

**Methods:**

In 2009, participants in the Commuting and Health in Cambridge study were sent questionnaires enquiring about sociodemographic characteristics and weekly time spent in active commuting. They were also invited to wear an accelerometer for seven days. Accelerometer data were used to compute the time spent in MVPA. Multiple regression models were used to examine the association between time spent in active commuting and MVPA.

**Results:**

475 participants (70% female) provided valid data. On average, participants recorded 55 (SD: 23.02) minutes of MVPA per day. For women, reporting 150 or more minutes of active commuting per week was associated with an estimated 8.50 (95% CI: 1.75 to 51.26, p = 0.01) additional minutes of daily MVPA compared to those who reported no time in active commuting. No overall associations were found in men.

**Conclusions:**

Promoting active commuting might be an important way of increasing levels of physical activity, particularly in women. Further research should assess whether increases in time spent in active commuting are associated with increases in physical activity.

## Highlights

► We studied a commuting population in Cambridge, UK in 2009. ► Time spent walking or cycling as part of commuting was assessed by questionnaire. ► Total moderate-to-vigorous physical activity (MVPA) was measured by accelerometry. ► We assessed the associations between active commuting and overall MVPA. ► Women reporting 150 min or more weekly active commuting achieved higher daily MVPA.

## Introduction

Physical activity is associated with a reduced risk of cardiovascular and other chronic diseases (Department of Health, 2011; Lee and Skerrett, 2001) and physical activity of longer duration and higher intensity appears to have greater benefits for health (Haskell et al., 2007). Whilst participation in recreational activity in the last few decades appears to have remained relatively stable (Brownson et al., 2005) or even increased (Stamatakis et al., 2007), occupational, transport-related and incidental physical activity has declined in many developed countries (Brownson et al., 2005). Cycling and brisk walking have been recommended as suitable moderate-to-vigorous physical activities (MVPA) (Haskell et al., 2007) since they can be incorporated into many people's daily routines and might therefore be more easily adopted and maintained than other forms of physical activity. Walking is a particularly accessible form of active travel (Ogilvie et al., 2007), while cycling can substitute for car trips of up to several miles (Department of Health and Department for Transport, 2010). Recent studies have shown that walking and cycling for travel are specifically associated with reduced cardiovascular risk and mortality and improved physical fitness in adults (Barengo et al., 2004; de Geus et al., 2009; Matthews et al., 2007).

Among developed countries, the prevalence of active travel for any purpose is highest in European countries (13 to 37%) and lowest in the US (3%) (Bassett et al., 2008). The prevalence of active travel to work (active commuting) in England and Wales in 2001 was 14% (Census, 2001) and is generally no higher in most other developed countries (Badland et al., 2007; Bassett et al., 2008). Previous studies suggest an association between active commuting and overall physical activity (Wener and Evans, 2007; Sisson and Tudor-Locke, 2008; Villanueva et al., 2008). However, these studies either used imprecise measures of physical activity such as step counts, which provide no information on intensity of activity (Wener and Evans, 2007; Villanueva et al., 2008), or were conducted in specific population groups such as university students (Sisson and Tudor-Locke, 2008; Villanueva et al., 2008). Furthermore, most previous studies have categorised commuting behaviour using the main mode of travel, i.e. travel by car, public transport, cycling or walking (Badland et al., 2007; Wener and Evans, 2007; Villanueva et al., 2008; Gordon-Larsen et al., 2009). These categories assign a single main mode to each trip and do not capture any combinations of travel modes used. For example, a commuter using public transport might have engaged in some walking or cycling at either end of the trip. Measures of time spent in walking and cycling as part of such trips would allow the associations with overall MVPA to be assessed more accurately. This is important because current guidance on the economic appraisal of the health benefits of walking and cycling relies on modelling to estimate the changes in overall physical activity associated with increases in walking and cycling because of a lack of empirical data on this relationship (Cavill et al., 2008).

Using data collected in the Commuting and Health in Cambridge study (Ogilvie et al., 2010), we used objectively measured physical activity data and detailed self-reported data on commuting behaviour to investigate whether time spent in active commuting was associated with MVPA in a large sample of working adults living in a mixture of urban and rural locations.

## Methods

The Commuting and Health in Cambridge study is designed to investigate travel behaviour, physical activity and health in adults who travel to work. The study design and participant recruitment have been described previously (Ogilvie et al., 2010). In brief, in 2009 we recruited 1164 men and women who were over 16 years of age, travelled to work in Cambridge and lived within a radius of approximately 30 km from the city centre through a predominantly workplace-based recruitment strategy. Between May and November 2009, participants completed a questionnaire that incorporated the Recent Physical Activity Questionnaire (RPAQ) (Besson et al., 2010), a four-week recall instrument to assess domain-specific physical activity closely based on the previously-validated EPAQ2 questionnaire (Wareham et al., 2002); a seven-day retrospective record of travel to and from work adapted from one used previously and shown to have acceptable test–retest reliability (Shannon et al., 2006); and a variety of sociodemographic and psychological items. Of those participants who agreed to wear an activity monitor, a random sample was invited to wear an accelerometer for seven days to have their physical activity objectively measured. The study was approved by the Hertfordshire Research Ethics Committee (reference number 08/H0311/208) and all participants provided written informed consent.

### Measures

#### Outcome — physical activity

Physical activity was objectively measured using the Actigraph accelerometer (models GT1M and GT3X) which is an extensively validated, small lightweight device that provides detailed information about the intensity, frequency and duration of physical activity (Ekelund et al., 2006). Participants were asked to wear the Actigraph on the right hip using a waist band during waking hours for seven consecutive days. Actigraphs were returned with the questionnaires by post. A recording of at least 10 h of data was defined as a valid day, and participants were included in the analysis if their device contained at least three valid days of recording. Actigraph data were processed using the MAHUffe program (available from http://www.mrc-epid.cam.ac.uk). Time spent in MVPA was defined as the average number of minutes per day in which > 1952 accelerometer counts were recorded (Freedson et al., 1998).

#### Exposure — time spent in active commuting

Commuting behaviour was assessed using the seven-day retrospective record of travel to and from work described above (Shannon et al., 2006). For each day of the week, participants were asked to report whether they travelled to work, the times at which they started and finished work, and the modes of travel used. They were also asked to report the usual number of minutes of walking and minutes of cycling involved in their journey to and from work, if any. Weekly times spent walking and cycling to and from work was calculated by multiplying the number of occasions by the usual number of minutes for each behaviour, and then summed to produce the weekly time spent in active commuting (walking and cycling combined). Owing to the large number of zero values, weekly time spent walking, cycling and in active commuting were categorised into three groups: none, less than 150 minutes, and 150 minutes or more. The three summary categorical exposure variables were included in separate models to explore possible differential associations with overall physical activity.

#### Covariates

Participants reported their gender, date of birth, height, weight and highest educational qualification, the presence of children under 16 years in the household, housing tenure, the distance between home and work, the type and amount of physical activity involved in their work, and their access to a car and to a bicycle. Age was calculated based on date of birth and date of completion of the questionnaire. Body mass index (BMI) was calculated as weight (in kilograms) divided by height squared (in metres). Urban–rural status of the home location was defined using the Urban and Rural Classification (Bibby and Shepherd, 2004) and based on the Census output area of their home postcode. The accumulated time that each participant had worn the Actigraph (wear time) was derived using MAHUffe.

#### Analysis

We summarised participant characteristics by gender using means and standard deviations for age and BMI, and frequencies and percentages for other variables. T-tests or chi-squared tests were used to assess differences in time spent walking, cycling and in active commuting according to whether valid Actigraph data were provided or not. Because of the differences in levels of MVPA between men and women and the documented differences in the prevalence of walking and cycling by gender (Ransdell et al., 2004), we conducted analyses separately for men and women as well as using the entire sample. Linear regressions were carried out to quantify the associations between (i) time spent walking, (ii) time spent cycling and (iii) time spent in active commuting (walking and cycling) and MVPA. In each model, variables representing participant characteristics were entered into the final models if they were significant at p < 0.1 in univariate analysis. Final models were also adjusted for accelerometer wear time. All analyses were performed using Stata version 11.0 (STATA Corp., College Station, Texas, USA).

## Results

A total of 714 participants were invited to wear an Actigraph. Of these, 486 (68.1%) provided usable physical activity data, of whom 475 (66.5% of those invited) also provided valid data on time spent in active commuting. These participants were included in our analysis. The characteristics of included participants and members of the wider study population who completed a questionnaire (n = 1164) are compared in Table 1 and the characteristics of the men and women included in the analysis are compared in Table 2. Participants were relatively active, recording an average of about 55 minutes of daily MVPA, and the majority (n = 338, 69.5%) were women.

Table 3 summarises the simple and adjusted associations between daily minutes of MVPA and weekly walking, cycling, and active commuting time respectively in men, women and the entire sample. In adjusted models, there was some evidence of an association between time spent walking to or from work and MVPA. In men, walking for 1–149 min per week was associated with achieving an estimated 13.97 (95% CI: 0.5 to 27.5, *p* = .04) additional minutes of daily MVPA compared with those who reported no walking on the journey to or from work. In women, walking to or from work for 150 minutes or more per week was associated with achieving an estimated 15.64 (95% CI: 6.6 to 24.7, *p* = .001) additional minutes of daily MVPA. There was no association between time spent in cycling to or from work and MVPA in men or women. Total time spent in active commuting was not associated with MVPA in men, but for women, spending 150 min or more per week in active commuting was associated with achieving an estimated 8.5 (95% CI: 1.8 to 15.3, *p* = .01) additional minutes of daily MVPA.

## Discussion

We have described the association between active commuting and objectively measured MVPA in a relatively active population in Cambridge, UK. The results of our adjusted analyses demonstrate that engaging in 150 min or more of active commuting per week contributes a significant amount of MVPA for women, resulting in about nine additional minutes of daily MVPA compared to women whose journey did not involve active commuting. This accounts for about 16% of the achieved daily MVPA in this population and about 30% of the recommended minimum level of MVPA.

The relatively high level of overall MVPA observed in this study may partly reflect the high level of leisure-time physical activity to be expected in a sample of relatively high socioeconomic status (Gidlow et al., 2006). On the other hand, the prevalence of engagement in any active commuting in our study (65%) was also high. Cambridge is not representative of UK cities in this respect: it stands out as Britain's leading ‘cycling city’, given that the proportion of cycling commuters in the 2001 Census was 28% in Cambridge City, 5% in the surrounding areas and 3% in England as a whole (Census, 2001). Previous studies have shown that using public transport can involve a substantial amount of walking, so that commuters who use public transport tend to walk more than those who travel by car (Villanueva et al., 2008; Wener and Evans, 2007). Unlike those studies, our analysis assessed the direct association between the specific quantities of active commuting and overall MVPA regardless of the main mode of travel for commuting. We found some evidence for an association between time spent walking to and from work and MVPA in men (for those walking for 1–149 min/wk) and stronger evidence in women (for those walking ≥ 150 min/wk).

Another US study reported that university students who cycled to campus accumulated more MVPA than those who used motorised modes (Sisson and Tudor-Locke, 2008). We found no association between time spent cycling and MVPA in our analysis. This probably reflects the different approaches taken to measuring MVPA in the two studies. Sisson and colleagues combined accelerometer-derived MVPA time with participant-logged cycling time to compensate for the underestimation of cycling-associated MVPA by accelerometer (Corder et al., 2007), while we have used the accelerometer-derived MVPA alone. Previous studies suggest that women tend to do less vigorous activity and more moderate-intensity activity than men, for example preferring walking to cycling (Ransdell et al., 2004). Indeed, commuter walking was more prevalent in women and commuter cycling was more prevalent in men in our sample, despite a high proportion of cyclists in both genders. Men who cycle may also cycle more than women for purposes other than commuting, either to reach non-work destinations or for leisure. The MVPA associated with these other types of cycling is also likely to have been underestimated by accelerometry. We used walking, cycling and combined active commuting time as exposure variables to fit separate regression models. Out of all three associations tested, we found the strongest to be that between MPVA and time spent walking. The association remained (but was lessened) when walking and cycling times were combined and disappeared when cycling time alone was used as the exposure variable. This loss of statistical significance suggests an underestimation of the cycling-associated MVPA, which could explain our finding of a lack of association between the time spent in active commuting and achieved MVPA in men.

### Strengths and limitations of the study

The main strengths of this study are the large sample and the precision of the measurements of the main exposure and outcome variables. We used self-reported weekly time spent in active commuting instead of classifying participants according to a predominant or main mode of travel to work. This allowed walking and cycling to be captured when used in combination with other modes of travel. There is little agreement on how cycling should be measured (Yang et al., 2010). Consequently, inconsistent measures of active commuting have been reported in previous studies with little or no information on their validity and reliability. We measured physical activity objectively by accelerometer. Nevertheless, the measurement of cycling by hip-worn accelerometer underestimates lower limb movements. The physical activity involved in activities such as cycling may be better captured using other devices such as the Actiheart (http://www.bio-lynx.com/actiheart.htm) or using an accelerometer worn on the ankle. However, neither of these alternative methods is likely to be as acceptable to participants in free-living population studies.

Out study sample was relatively homogeneous, being drawn from a working population mainly recruited through workplaces in Cambridge, and Cambridge itself has an unusually well established cycling culture. Furthermore, the sampling strategy (which was predominantly based on opt-in recruitment through workplaces) means that our sample cannot be assumed to be representative of Cambridge commuters. These considerations limit the generalisability of our findings. Furthermore, our findings are based on cross-sectional analyses; we therefore have no basis for suggesting a causal association between taking up active commuting and a higher level of MVPA in women. The association we have reported should therefore be corroborated in other settings and using longitudinal designs incorporating robust measures of MVPA which adequately capture both walking and cycling behaviours. In addition, the social distribution of the association needs to be studied in other samples with greater sociodemographic heterogeneity.

## Conclusion

In this study of a relatively active sample of working adults, men were more likely than women to report cycling, and women were more likely than men to report walking, as part of the journey to and from work. Active commuting was associated with higher levels of overall MVPA, at least in women, independently contributing a quarter of the recommended overall level of MVPA. Further validation studies are needed to establish suitable measures of time spent walking and cycling and objective methods of capturing cycling in large epidemiological studies. Further research investigating a possible casual relationship between active commuting and overall physical activity is needed, incorporating improved objective measures of cycling, to gain a clearer understanding of whether taking up active commuting results in an increase in overall physical activity.

## Conflict of interest

The authors declare that there are no conflicts of interests.

## Figures and Tables

**Table 1:** Descriptive characteristics of participants in the Commuting and Health in Cambridge study by whether or not they provided valid MVPA and active commuting data.

	Overall		Did not provide valid MVPA and active commuting data (n = 689)		Provided valid MVPA and active commuting data (n = 475)		*p*
	N	%	N	%	N	%	
Age mean (SD) years (n = 1163)^a^	42.3	(11.4)	41.7	(11.8)	43.2	(10.9)	0.03
BMI mean (SD) kg/m^2^ (n = 1145)^a^	24.5	(4.2)	24.5	(4.4)	24.5	(3.9)	0.89
Difficulty walking (n = 1161)^a^							
No	1143	(98.5)	676	(98.4)	467	(98.5)	0.87
Yes	18	(1.6)	11	(1.6)	7	(1.5)	
Education (n = 1155)^a^							
Degree or higher	834	(72.2)	497	(72.9)	337	(71.3)	0.55
Less than degree	321	(27.8)	185	(27.1)	136	(28.7)	
Child under 15 in household (n = 1164)							
Yes	345	(29.6)	194	(28.2)	151	(31.8)	0.18
No	819	(70.4)	495	(71.8)	324	(68.2)	
Home ownership (n = 1164)							
Home owner	840	(72.2)	462	(67.0)	378	(79.6)	< 0.001
Rented or other	324	(27.8)	227	(33.0)	97	(20.4)	
Distance from home to work (n = 1162)^a^							
5 km or less	454	(39.1)	349	(50.8)	105	(22.1)	< 0.001
5.01–10 km	221	(19.0)	114	(16.6)	107	(22.5)	
10.01–20 km	183	(15.8)	85	(12.4)	98	(20.6)	
Over 20 km	304	(26.2)	139	(20.2)	165	(34.8)	
Car ownership (n = 1164)							
No car	175	(15.0)	127	(18.4)	48	(10.1)	< 0.001
One car	526	(45.2)	334	(48.5)	192	(40.4)	
More than one car	463	(39.8)	228	(33.1)	235	(49.5)	
Access to bicycle (n = 1156)^a^							
Yes	974	(84.3)	580	(84.9)	394	(83.3)	0.46
No	182	(15.7)	103	(15.1)	79	(16.7)	
Home location (n = 1163)^a^							
Urban	767	(66.0)	504	(73.3)	263	(55.4)	< 0.001
Rural	396	(34.1)	184	(26.7)	212	(44.6)	
Physical activity level at work (n = 1162)^a^							
Sedentary job	935	(80.4)	549	(79.7)	386	(81.6)	0.42
Non-sedentary job	227	(19.5)	140	(20.3)	87	(18.4)	
Reported any walking to and from work (n = 1149)^a^							
Yes	327	(28.5)	207	(30.7)	120	(25.3)	0.04
No	822	(71. 5)	467	(69.3)	355	(74.7)	
Reported any cycling to and from work (n = 1154)^a^							
Yes	595	(51.6)	378	(55.7)	217	(46.7)	0.001
No	559	(48.4)	301	(44.3)	258	(54.3)	
Reported any active commuting (n = 1164)							
Yes^b^	842	(72.3)	529	(76.8)	313	(65.9)	< 0.001
No	322	(27.7)	160	(23.2)	162	(34.1)	

Data are reported as N and % unless otherwise specified. Data were collected between May and November 2009 in Cambridge, UK.

a n < 1164 due to missing data.

b Participant reported walking, cycling or both as part of their journey to and from work.

**Table 2:** Descriptive characteristics of participants providing valid MVPA data in the Commuting and Health in Cambridge study by gender.

	Men		Women		*p*
	N	%	N	%	
Age mean (SD) years (n = 475)	44.1	(10.1)	42.7	(11.2)	0.22
BMI mean (SD) kg/m^2^ (n = 467)^a^	25.0	(3.1)	24.3	(4.2)	0.07
Difficulty walking (n = 474)^a^					
No	142	(97.9)	325	(98.8)	0.48
Yes	2	(2.1)	4	(1.2)	
Education (n = 473)^a^					
Degree or higher	118	(81.4)	219	(66.8)	0.001
Less than degree	27	(18.6)	109	(33.2)	
Child under 15 yr in household (n = 475)					
Yes	63	(43.5)	88	(26.7)	< 0.001
No	82	(56.5)	242	(73.3)	
Home ownership (n = 475)					
Home owner	117	(80.7)	261	(79.1)	0.69
Rented or other	28	(19.3)	69	(20.1)	
Distance from home to work (n = 475)					
5 km or less	36	(24.8)	69	(20.9)	0.50
5.01–10 km	36	(24.8)	71	(21.5)	
10.01–20 km	29	(20.0)	69	(20.9)	
Over 20 km	44	(30.3)	121	(36.7)	
Car ownership (n = 475)					
No car	20	(13.8)	28	(8.5)	0.21
One car	56	(38.6)	136	(41.2)	
More than one car	69	(47.6)	166	(50.3)	
Access to bicycle (n = 473)^a^					
Yes	130	(90.3)	264	(80.2)	0.01
No	14	(9.7)	65	(19.8)	
Home location (n = 475)					
Urban	80	(55.2)	183	(55.5)	0.96
Rural	65	(44.8)	147	(44.5)	
Physical activity level at work (n = 473)^a^					
Sedentary job	119	(82.6)	267	(81.2)	0.70
Non-sedentary job	25	(17.4)	62	(18.8)	
Time spent walking to and from work (n = 475)					
0 min/wk	119	(82.1)	236	(71.5)	0.01
1–149 min/wk	14	(9.7)	70	(21.2)	
≥ 150 min/wk	12	(8.3)	24	(7.3)	
Time spent cycling to and from work (n = 475)					
0 min/wk	59	(40.7)	199	(60.3)	< 0.001
1–149 min/wk	31	(21.4)	59	(17.9)	
≥ 150 min/wk	55	(37.9)	72	(21.8)	
Time spent in active commuting^b^ (n = 475)					
0 min/wk	37	(25.5)	125	(37.9)	0.003
1–149 min/wk	41	(28.3)	104	(31.5)	
≥ 150 min/wk	67	(46.2)	101	(30.6)	
MVPA mean (SD) (min/day) (n = 475)	59.45	(23.4)	53.03	(22.6)	0.005

Data are reported as N and % unless otherwise specified. Data were collected between May and November 2009 in Cambridge, UK.

a n < 475 due to missing data.

b Time spent in active commuting is the sum of time spent walking to and from work and the time spent cycling to and from work for each participant.

**Table 3:** Associations between weekly time spent in walking, cycling, or both to and from work and overall daily MVPA.

Main exposure	MVPA (men, n = 141)				MVPA (women, n = 320)				MVPA (total, n = 453)			
	Unadjusted model		Adjusted model^a^		Unadjusted model		Adjusted model^b^		Unadjusted model		Adjusted model^c^	
	Regression coefficient (95% CI)	*p*	Regression coefficient (95% CI)	*p*	Regression coefficient (95% CI)	*p*	Regression coefficient (95% CI)	*p*	Regression coefficient (95% CI)	*p*	Regression coefficient (95% CI)	*p*
Model 1: time spent walking to and from work (reference: none)												
Walking 1–149 min/wk	12.28 (− 0.7 to 25.3)	0.06	13.97 (0.5 to 27.45)	0.04	5.74(− 0.3 to 11.8)	0.06	5. 72 (− 0.1 to 11.5)	0.05	5. 35 (0.2 to 10.9	0.06	6.50 (1.1 to 11.9)	0.02
Walking ≥ 150 min/wk	11.73 (− 3.4 to 26.9)	0.13	13.27 (− 1.7 to 28.2)	0.08	15.74 (6.3 to 25.1)	0.001	15.64 (6.6 to 24.7)	0.001	14.22 (6.2 to 22.2)	0.001	14.33 (6.5 to 22.2)	0.001
Model 2: time spent cycling to and from work (reference: none)												
Cycling 1–149 min/wk	− 3.69 (− 14.2 to 6.8)	0.49	− 0.65 (− 11.0 to 9.7)	0.90	− 0.97 (− 7.7 to 5.8)	0.78	− 4.78 (− 11.8 to 2.3)	0.18	− 0.26 (− 6.0 to 5.5)	0.93	− 3.04 (− 8.7 to 2.7)	0.30
Cycling ≥ 150 min/wk	− 6.83 (− 15.6 to 2.0)	0.13	− 7.51 (− 16.6 to 1.56)	0.10	4.12 (− 2.1 to 10.3)	0.19	− 1.05 (− 8.1 to 6.0)	0.77	1.65 (− 3.4 to 6.7)	0.52	− 2.85 (− 8.0 to 12.3)	0.28
Model 3: time spent in active commuting (reference: none)												
Active commuting 1–149 min/wk	1.40 (− 9.3 to 12.1)	0.80	3.54 (− 6.6 to 13.7)	0.49	5.36 (− 0.6 to 11.3)	0.08	4.51 (− 1.8 to 10.8)	0.16	4.68 (− 0.6 to 10.0)	0.08	3.08 (− 2.2 to 8.4)	0.25
Active commuting ≥ 150 min/wk	− 1.80 (− 11.4 to 7.9)	0.71	− 2.07 (− 11.5 to 7.4)	0.67	10.39 (4.5 to 16.3)	0.001	8.50 (1.8 to 15.3)	0.01	7.45 (2.4 to 12.5)	0.004	4.1 (− 1.2 to 9.4)	0.13

Data were collected between May and November 2009 in Cambridge, UK.

a Adjusted for age, car ownership, access to bicycles and accelerometer wear time.

b Adjusted for BMI, distance from home to work, car ownership, physical activity level at work and accelerometer wear time.

c Adjusted for gender, BMI, car ownership, physical activity level at work and accelerometer wear time.
